# A single center observational study of the incidence, frequency and timing of critical care physiotherapy intervention during the COVID-19 pandemic

**DOI:** 10.1177/1751143721991060

**Published:** 2022-08

**Authors:** Jessica Rich, Mark Coman, Alison Sharkey, Daniel Church, Jessica Pawson, Amanda Thomas

**Affiliations:** 1 Physiotherapy Department, Royal London Hospital, London, UK; 2 Acute Critical Care Unit, Royal London Hospital, London, UK; 3 After Trauma Team, Royal London Hospital, London, UK; 4 Physiotherapy Department, Royal London Hospital, London, UK; 5 Critical Care Outreach Team, Royal London Hospital, London, UK

**Keywords:** COVID-19, critical care, physiotherapy

## Abstract

**Introduction:**

The recent COVID-19 pandemic saw many patients admitted to an intensive care setting and requiring mechanical ventilation. The NHS increased their critical care beds which included expanding the amount of staff. Physiotherapists were a key part of this and were required to complete numerous interventions within the COVID critical care setting throughout the pandemic. Our aim was to collect the incidence and frequency of physiotherapy interventions performed during the COVID-19 pandemic in a critical care setting.

**Method:**

Data was collected across all critical care beds at the Royal London Hospital for an eight-week period between March- April 2020. We retrospectively collected physiotherapy interventions for example, endotracheal suctioning and functional rehabilitation for every patient in the critical care setting. The Chelsea Critical Care Physical Assessment Tool (CPAx) scores were also obtained for patients on ACCU admission and discharge.

**Results:**

A total of 213 patients were included in the sample, 163 COVID-19 positive and 50 COVID-19 negative. Recorded sessions included secretion management (821), weaning (271), rescue therapy (82) and functional rehab (534) across the eight-week period. The mean CPAx score on admission to ACCU for the entire sample was 9/45 points. On discharge that score had improved to 25/45 points.

**Conclusion:**

This unique project has enabled us to report on the critical care physiotherapy interventions provided during the COVID 19 pandemic. This interesting data on frequency and timing of interventions may be useful to plan future relocation staffing plans and optimal allocation of care.

## Introduction

The United Kingdom SARS-CoV-2 index case was declared in York on the 31st January, 2020. The ensuing COVID-19 pandemic reached its peak in the UK in April, 2020, requiring a rapid response from the National Health Service (NHS). This response included expansion of critical care services and redeployment of staff groups to facilitate enhanced capacity. Staff redeployment highlighted the need for rapid upskilling and training. Training was guided by the concomitant increase in published practice recommendations for staff groups contributing to critical care expansion. COVID-19 practice recommendations for Physiotherapists in acute hospital settings first appeared in late March, 2020.^1,2^ These recommendations were based on best available evidence and expert consensus from countries yet to be significantly affected by the virus,^1^ and the specific experience of Physiotherapists from northern Italy who had been experiencing an exponential growth of COVID-19 infections since early February, 2020.^2^

Physiotherapy clinicians within the critical care setting quickly recognized disparity between these practice recommendations and the presentation-based needs of our COVID-19 population, both within and outside critical care. Subsequently, we proposed to capture the Physiotherapy service delivered to our COVID-19 critical care cohort to determine incidence, frequency and timing of interventions. This data may support future respiratory pandemic curricula, by identifying interventions which are likely to be employed early, compared to those which may be required following the peak. In addition, this data adds to the global identification of Physiotherapy requirements for patients with COVID-19 related illness and may inform future practice recommendations.

## Methods

*Design and setting:* This was a single center observation completed at the Adult Critical Care Unit (ACCU) of the Royal London Hospital (RLH), London, United Kingdom. Data collection ran from 1st April to 31st May 2020. The ACCU at RLH usually hosts 44 critical care beds, occupied by medical, surgical and trauma patients. In response to COVID-19, critical care bed capacity was expanded to 90 beds catering for both COVID-19 positive and negative patients. Patient groups were cared for in separate clinical areas as the ACCU physically expanded into theatre recovery, pediatric intensive care and renal high dependency.

Forty-two Physiotherapists (Critical Care Physiotherapy specialists and redeployed staff) were available to provide a seven day service creating an average Physiotherapist to bed ratio of 1:5. Core services were delivered between 8am and 6 pm, with overnight on-call from 6 pm to 8am. The early pandemic response involved practical based learning for redeployed staff. All Physiotherapy staff were fit tested and provided with Personal Protective Equipment (PPE) training.

Physiotherapists used clinical reasoning to review patients that would benefit from physiotherapy input. Physiotherapy reasoning was supported by a Standard Operating Procedure (SOP) for Critical Care. The SOP described safe practice for aerosol generating procedures common in respiratory physiotherapy and a prioritization tool to support clinical decisions in relation to staffing capacity. For example, patients with urgent respiratory Physiotherapy needs were prioritized over patients with physical rehabilitation needs alone.

*Participants:* All patients occupying an ACCU bed over the data collection period were included, irrespective of COVID-19 status.

*Data collection:* A data collection tool was created *a-priori* including hospital number, date of birth, admission and discharge from critical care. For the purpose of the data collection, Physiotherapy interventions were classified as five categories demonstrated in Table 1: – Physical Assessment and Limb Care; Secretion management; Weaning; Rescue Therapies and Functional Rehabilitation. “Physical Assessment and Limb Care” was classified as a therapy intervention since it is the primary method of determining therapy needs in the ACCU environment. The time required to complete a thorough physical assessment of therapy needs justifies establishing this category.

**Table 1.** List of physiotherapy interventions.

Physiotherapy Interventions	
Secretion Management	Suctioning *(The use of both hard and soft catheters to clear secretions, either through open or closed circuits)* Nasopharyngeal airway/NPA placement *(Nasal pharyngeal airway placement to allow ease of regular suctioning)* Ventilator hyperinflation/VHI *(Manually and temporarily changing Ventilator settings to produce a hyperinflation phenomenon to clear airway secretions)* Repositioning *(Clinically reasoning an indication for a change in body position, either actively or passively to assist with postural drainage)* Nebulisers *(Provision of nebulisers to aid airway clearance and advice re: timing and frequency of administration)* Manually assisted cough/MAC *(Providing physical assistance to the abdomen & chest wall to mimic muscle activity to produce an effective cough)* Manual techniques *(Provision of physical techniques including percussion & vibrations of the chest wall to aid secretion clearance)*
Weaning	Ventilator weaning *(Participating in Multi-Disciplinary Team (MDT) decisions regarding ventilator weaning and actively changing ventilator modes & settings as tolerated)* Tracheostomy wean *(Providing specialist tracheostomy assessment and actively developing bespoke weaning programs, ranging from ventilated tracheostomy airways to decannulation)*
Rescue Therapies	Self proning *(Providing advice and practice to allow a patient to independently adopt a prone position to optimise ventilation)* Prone *(Providing physical assistance to place a patient in an effective prone position to optimise ventilation)*
Functional Rehabilitation	Sitting on the edge of the bed/SOEOB *(Providing assistance or supervision to support a patient to move from a lying to seated position in bed, and maintaining this position as able)* Sitting out in a chair/SOOBIC *(Providing assistance or supervision to support a patient to move from the bed to safely sit in a chair. This could also include the use of assistive automated or static devices e.g. hoists.)* Mobilising *(Mobility interventions including standing practice, balance training, gait education and functional mobility)*

Physical morbidity of the sample was measured using The Chelsea Physical Assessment tool (CPAx) at admission and discharge. CPAx is a measure of physical morbidity in general critical care cohorts. There are 10 CPAx domains with a score from zero (complete dependence) to five (complete independence).^3^ As we were unable to measure the handgrip strength domain during the pandemic, the maximum CPAx score anticipated was 45, rather than 50.

Data was extracted from the electronic patient records and retrospectively entered within a password protected spreadsheet locally stored in compliance with General Data Protection Regulation (GDPR 2018). A descriptive analysis of the data was completed outlining incidence, frequency and temporal relationships of intervention delivery.

*Ethical Considerations:* Ethical approval and patient consent were not required as the project was registered as a service evaluation by the clinical effectiveness unit at The Royal London Hospital (project 11,153). There was no deviation from usual care for any patient.

## Results

213 patients were included in the sample, 163 (76%) COVID-19 positive and 50 (23%) COVID-19 negative. The demographics of the sample are presented below (Table 2).

**Table 2.** Demographic information.

	COVID-19 positive patients	COVID-19 negative patients
Patient number *(n)*	163	50
Age *(M ± SD)*	56 ± 14.8	50 ± 17.5
Male/female *(%)*	118/45 *(72/28)*	34/16 *(70/30)*
Length of stay (*M ± SD)*	12.9 ± 11.5	7 ± 6.6
Mortality *(%)*	53 *(32.5%)*	4 *(8%)*
*M/F*	*44/7*	*4/0*

**Figure 1.**
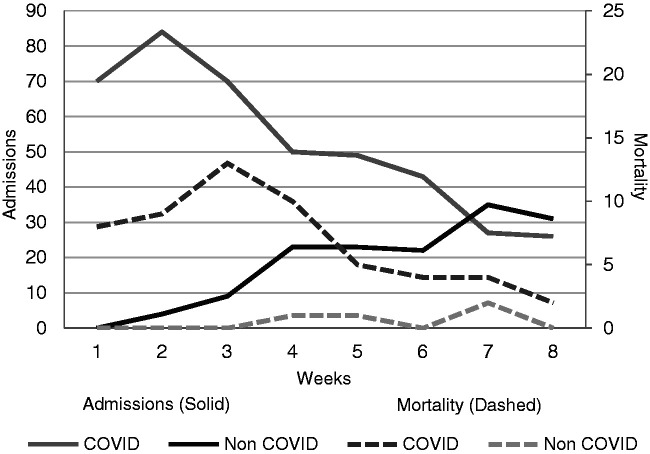
ACCU admissions and mortality per week.

COVID-19 positive patients had a mean age of 56 ± 14.8 years compared to 50 ± 17.5 years in the negative group. Males **comprised** greater than 70% of each cohort. Mean length of stay (LOS) for COVID-19 positive patients was 12.9 ± 11.5 days compared to 7 ± 6.6 days in the negative group. There was higher mortality in the COVID-19 positive group (32.5%; *n* = 53) compared to negative patients (8%; *n* = 4). Figure 1 illustrates the distribution of ACCU mortality. While 53 patients died across the period, the peak mortality (12 deaths) occurred during week three. It also demonstrates the number of COVID-19 positive and negative patients during this time period, the peak (86 patients) occurred at week two when COVID-19 positive patients reached a zenith. At the completion of data collection there were more COVID-19 negative (31 patients) than COVID-19 positive (27 patients).

**Figure 2.**
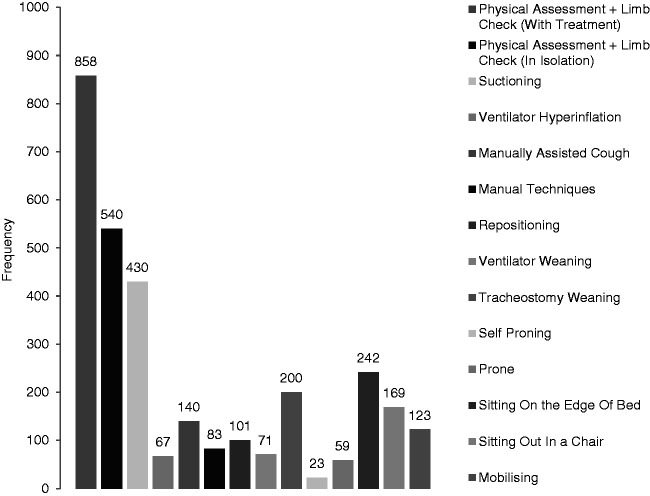
Intervention frequency distribution.

48% of the sample (*n* = 76) completed both an initial and discharge CPAx while in the ACCU, the remainder had incomplete data. The mean CPAx on admission to ACCU was 9/45 points, and 25/45 points on discharge. The mean admission COVID-19 positive CPAx was 9.1 with their COVID-19 negative counterpart being 10.5. On discharge COVID-19 positive patients demonstrated an average score of 24.3; vs. 28.9 for COVID-19 negative (Figure 2).

In total, 3106 physiotherapy interventions were delivered across the time period. Frequency per treatment is described in Figure 2. Physical Assessment and Limb Care was the most common intervention either in “isolation” where further treatment was not clinically indicated (540 occasions) or “with treatment” (858 occasions) where additional treatment techniques were clinically indicated. The frequency of “physical assessment and limb care” in isolation peaked during the second week of data collection, before gradually declining.

**Figure 3.**
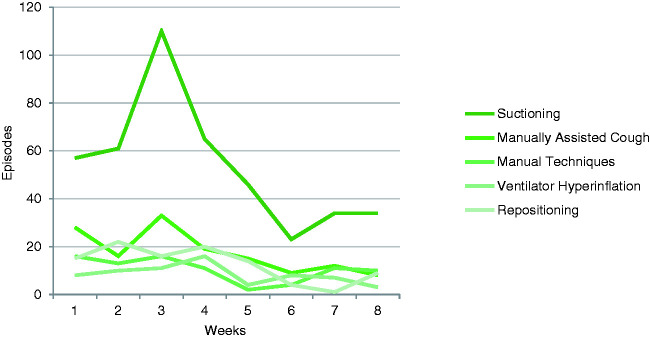
Frequency and distribution of secretion management techniques.

The most frequent secretion management intervention was suctioning (430 occasions), followed by positioning (101 occasions), assisted cough (140 occasions), ventilator hyperinflation (67) and manual techniques (83 occasions) and (Figure 3).

**Figure 4.**
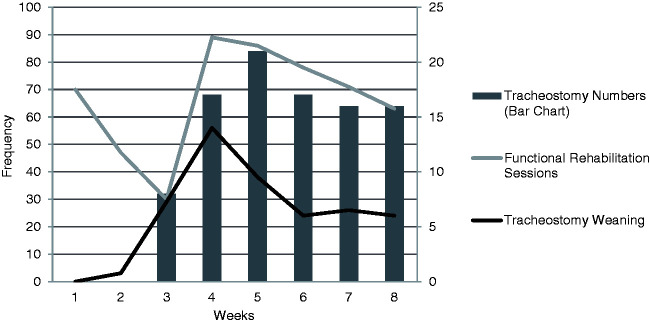
Frequency and distribution of tracheostomy numbers, functional rehabilitation sessions and tracheostomy weaning.

Weaning interventions were completed on 271 occasions. The distribution demonstrated an increase in tracheostomy weaning over the third, fourth and fifth week (Figure 4), while ventilator weaning was delivered more consistently throughout the period.

Rescue therapies were required on 82 occasions. The temporal requirement for these interventions were predominantly in the first two weeks. Rescue therapies were rarely indicated beyond weeks four and five.

Functional rehabilitation (FR) was required on 534 occasions, representing 242 SOEOB, 169 SOOBIC and 123 mobility interventions. The mean weekly incidence of FR was 67 ± 11.8 occasions. Figure 4 reveals the distribution of interventions was greatest in weeks four, five and six. In the first three weeks of data collection less than 26% of interventions were dedicated to FR compared to 36% at week six. The incidence of FR increased in parallel with tracheostomy insertion.

## Discussion

Our data collection period commenced at the peak of COVID 19 admissions to critical care within our hospital. Consequently our data describes of the incidence, frequency and temporal requirements for critical care physiotherapy over an eight week period immediately following the COVID-19 Pandemic peak and reflects the therapy needs of a group of patients admitted during this time period. While there are examples in the literature recommending the scope and standard of critical care Physiotherapy,^4–6^ including specific recommendations for respiratory Physiotherapy during the COVID-19 pandemic,^2^ there are no detailed descriptions of the interventions employed or the incidence, frequency or temporal requirements of those interventions. Descriptions of this nature are essential to inform workforce planning and training in the event of future pandemic responses For example, our data demonstrates in the first three weeks there was a greater requirement for physical assessment, rescue therapies and secretion management interventions, compared to weaning interventions and mobility activities. COVID-19 related mortality was also high during this early period, suggesting that acuity may have limited therapy intervention, despite the number of COVID-19 admissions being higher during this time frame. The first four weeks of data collection also recorded the highest incidence of proning interventions, which were rarely required in the last four weeks. The incidence of “physical assessment and limb care” alone during the first three weeks supports the premise that acuity was high and some interventions were contra-indicated.

In contrast, from week three patients began having a tracheostomy inserted for ventilator weaning. Consequently, there was a greater requirement for tracheostomy weaning interventions and a spike in suctioning events observed. The requirement for manually assisted coughing, manual techniques, ventilator hyperinflation and positioning declined after week three demonstrating the shift in therapy emphasis as patients progressed through their illness trajectory. Our data also demonstrates that tracheostomy insertion was associated with increased frequency of rehabilitation interventions. Activity interventions rose dramatically after the first three weeks and represented the greater percentage of therapy delivered over the last weeks of the project. Research has previously reported that 63% of patients with tracheostomy managed to sit out of the bed during their critical care stay but when these activities occurred within the temporal admission framework remains unknown.^7^

Some interventions remained static throughout the eight-week period demonstrating low stable weekly frequency. For example, ventilator weaning averaged nine occasions per week across the time period and showed little variation in comparison to other interventions. It is curious to appreciate that the end of our data collection period was associated with easing of lockdown restrictions in the United Kingdom which may be reflected in the increased COVID-19 negative admissions (predominantly trauma related) observed during weeks seven and eight. Many of the intervention frequencies captured in this project also demonstrate upward deflection in the last two weeks of the project, reflecting the requirement for physiotherapy interventions in the COVID-19 negative cohort.

Previous reports of critical care Physiotherapy in non-pandemic periods have focused on frequency of intervention delivery over the critical care admission, but have not included a temporal analysis. These studies have highlighted the proportion of Physiotherapy intervention related to rehabilitation activities. One study reported frequencies for all therapy interventions in 82 mixed medical and surgical patients over a three month period.^8^ These authors report a 55% incidence of functional rehabilitation activities during that time period, however the temporal characteristics of these interventions was not reported. Similarly, a report of 194 Physiotherapy treatment sessions over a six week period in a mixed medical surgical critical care described active rehabilitation in 51% of all sessions.^9^ The median time to commence active rehabilitation from critical care admission was reported as three days, but there was a large range (three to forty-three days). Another exploration of 327 physiotherapy episodes over four weeks in a mixed medical, surgical and trauma critical care reported a 54% incidence of rehabilitation intervention, without reporting temporal characteristics of intervention delivery.^10^

In the event of another respiratory pandemic, initial training for re-deployed critical care therapy staff should prioritize physical assessment of the critically ill patient, proning techniques and secretion management interventions. Tracheostomy interventions and functional rehabilitation should be approached at a time point closer to when these skills may be utilized in the pandemic progress. Interventions which maintain low incidence and stable frequency (e.g. ventilator weaning) could be omitted from training programs if there are sufficient critical care trained staff to deliver these interventions when required. Understanding the temporal requirement for critical care physiotherapy interventions during a pandemic may support targeted therapy training opportunities and increase the effectiveness of learning.

The findings of this evaluation should be viewed in light of several limitations. The rapidly evolving nature of the COVID-19 pandemic meant that the data collection tool was designed quickly with limited capacity for extensive planning or consideration of all factors which might be considered. We chose to capture manageable variables factoring projected staff sickness rates and the potential that staff could be rapidly returned to their original specialties, unknown patient volume and subsequent demand for our services. In the absence of this time constraint we may have considered recording ethnicity and comorbidities within our patient demographic to illustrate comorbid disease prevalence of our population. CPAx was recorded for less than half of our dataset. Compliance with weekly CPAx scoring could have been more consistent, with task prioritization, staff experience and familiarity limiting consistent scoring. In addition, the discharge CPAx did not always correlate with discharge from ACCU, since the score was calculated once per week. Increasing the frequency of CPAx scoring may have improved the percentage of scores available for analysis. Despite these limitations we were able to demonstrate (in a third of our sample) a 16point change in CPAx from admission to critical care discharge. The minimal clinically significant difference in burns patients CPAx has been reported as six points.^10^ It remains unknown what CPAx change score is significant within a COVID population.

Although there was a large volume of Physiotherapy intervention delivered during this project, it remains unknown whether the frequency of intervention was due to staffing capacity alone, or whether it was meeting the Physiotherapy needs of the patient. For example, United Kingdom guidelines recommend one Physiotherapist for every four critical care beds across a seven day working week,^11^ while the European society of intensive care medicine recommend a Physiotherapist for every five critical care beds.^12^ Since the staffing ratio achieved by redeployment achieved these targets, this report represents a description of a Physiotherapy service delivered with United Kingdom recommended staffing ratios. Although these staffing ratios influenced incidence and frequency of intervention delivery, it is difficult to establish whether outcomes were affected. An additional limitation of this project was the failure to record activity of the overnight physiotherapy service, to determine the effect of enhanced daylight services on out of hours emergency physiotherapy requirements.

Therapy staffing and the relationship to occupied critical care beds is rarely reported in the international literature. One observational study exploring critical care Physiotherapy practice in Australia and Scotland reported a mean ratio of (1: 5.6) Physiotherapist to ICU beds in ten Australian hospitals (minimum 1: 3; maximum 1: 8.5) and nine Scottish hospitals (mean 1: 6.7; minimum 1: 3.3; maximum 1: 10).^13^ Another report described the weekday service delivered to a 30-bed teaching hospital including five respiratory and three rehabilitation Physiotherapists to elicit a ratio of one Physiotherapist to 3.75 occupied beds.^8^ A further study explored the introduction of a critical care weekend service on physiotherapy interventions frequency.^14^ Increasing the weekend service from one to three staff increased the mean number of patients receiving a weekend intervention and the number of interventions, despite the therapy staff to patient ratio being 1:24. None of these investigations reported the link between therapy staffing and patient outcomes.

It is also important to recognize that the number of critical care interventions *delivered* during this project may not be a reflection of the intensity of Physiotherapy that was *required*. Other than clinical reasoning, there is no formal strategy to determine the ideal intensity of physiotherapy intervention in the patient with critical illness. Typically, therapy frequency is provided on the basis of capacity to deliver, rather than in relation to patient need. Although our staffing levels were temporarily enhanced for pandemic planning, it remains unclear whether the intervention frequency reported remained affected by staffing capacity. Future research should consider understanding how therapy intensity can be related to observed critical care patient needs. Other acute care disciplines have complexity scales which recommend therapy intervention input dependent on patient presentation.^15^ A similar scale for patients with critical illness would provide therapists with an understanding of each patient’s therapy needs (frequency and intensity) and the capacity to deliver that intensity should be reflected by staffing capacity.

Finally we acknowledge that statistical testing was not established for this report and observational outcomes may limiting its conclusions.

## Conclusion

This report describes the incidence, frequency and temporal requirement of critical care physiotherapy intervention during the COVID-19 pandemic peak in a teaching hospital when Physiotherapist to bed ratios were commensurate with recommendations in the United Kingdom. There was a temporal variation in the requirement for Physiotherapy intervention over the eight weeks. This data should enable future training for re-deployed therapy staff to be focused on interventions which are likely to be required during the initial clinical response, and subsequent graded delivery of training for interventions which are required at a later time point.

## Supplemental Material

sj-pdf-1-inc-10.1177_1751143721991060 - Supplemental material for A single center observational study of the incidence, frequency and timing of critical care physiotherapy intervention during the COVID-19 pandemic Click here for additional data file. Supplemental material, sj-pdf-1-inc-10.1177_1751143721991060 for A single center observational study of the incidence, frequency and timing of critical care physiotherapy intervention during the COVID-19 pandemic by Jessica Rich, Mark Coman, Alison Sharkey, Daniel Church, Jessica Pawson and Amanda Thomas in Journal of the Intensive Care Society

## Supplemental Material

sj-pdf-2-inc-10.1177_1751143721991060 - Supplemental material for A single center observational study of the incidence, frequency and timing of critical care physiotherapy intervention during the COVID-19 pandemic Click here for additional data file. Supplemental material, sj-pdf-2-inc-10.1177_1751143721991060 for A single center observational study of the incidence, frequency and timing of critical care physiotherapy intervention during the COVID-19 pandemic by Jessica Rich, Mark Coman, Alison Sharkey, Daniel Church, Jessica Pawson and Amanda Thomas in Journal of the Intensive Care Society
