# Supplementary material for: A single center observational study of the incidence, frequency and timing of critical care physiotherapy intervention during the COVID-19 pandemic
Source: J Intensive Care Soc. 2021 Feb 9;23(3):273–80. doi: 10.1177/1751143721991060 (PMC7873622; doi:10.1177/1751143721991060)
Supplement: Supplementary material [file sj-pdf-2-inc-10.1177_1751143721991060.pdf]

Reviewer: 1

Introduction:

- page 3 line 15: recommend omitting the word 'Fortunately' – **This has been taken out.**

Methods:

- page 4 line 5: semicolon should be a comma – **This has been changed**

- page 4 line 18: 'critical care trained' - consider rewording 'trained in critical care' – **This has been changed**

- page 4 line 30: 'supported' should be 'support' – **This has been changed**

- page 4 lines 48 - 53: These sentences need more clarity: "Assessment of disability and exposure are inclusive of peripheral joint integrity and muscle length. Therefore, "assessment and limb care" was classified as an intervention strategy when it was delivered alone, i.e., additional interventions not required. " Although I understand this (as a physio), I think you need to make it clearer for a multidisciplinary audience. You're basically saying you counted assessment-alone as an intervention, but I think it needs more explaining here (and why that's a reasonable thing to do, perhaps in terms of time required for a thorough assessment) – **Agreed this has now been changed for further clarity**

- page 5 line 12: "The Chelsea Physical Assessment (CPAx) score at critical care admission and discharge was recorded." (Reorganise this sentence to read "was recorded at admission and discharge". Even better to open this paragraph with a sentence about measuring physical function.- **This has been changed**

Results:

- page 5 line 49: consider "comprised" rather than "represented". Also missing full stop after cohort – **This has been changed.**

- page 6 line 3: "57 patients died, the peak (12 deaths) occurred during week three. " This sentence could be reworded to flow better: "While 53 patients died across the period, the peak mortality (12 deaths) occurred during week three." – **This has been changed**

- page 6 line 15: don't need 2 decimal places for the CPAx score here – **This has been changed**

Discussion:

- page 7 line 38: highest number (instead of most number) – **This has been changed**

- page 8 line 26: Tracheostomy doesn't need to be capitalised – **This has been changed**

- page 10: 'Therapy staffing' paragraph. Please consider significantly condensing this paragraph. Although it's interesting, you need to directly link it with staffing in a pandemic context, rather than have the information sit alone here without any direct link – **This has now been condensed**

- page 11 lines 9 - 10: "We might conclude that our sample had a change more than six points and that the reported intervention frequency improved physical outcomes during the critical care admission" . I would suggest removing this statement, as this type of study cannot draw this conclusion (natural improvement is of course the elephant in the room here!) – **This has been removed**

- page 11 lines 11 - 17: "It remains unknown what the clinically significant CPAx admission to discharge change score is in a mixed COVID-19 positive and negative cohort, although the scores reported do provide information regarding the physical morbidity of the sample throughout the time period." - not clear to me what value this paragraph adds - the reader knows that COVID-19 is a novel process, but there is no reason to think that CPAx would not be valid in this context. Suggest omitting these sentences (your discussion is very long anyway, so this would be a way to reduce word count here) – **This has been removed**

- page 11 lines 49 - 53: "A Physiotherapy service was provided to COVID-19 positive and negative patients without distinction guided by a prioritization tool and local operating procedure." - remove these sentences, just repetition and not necessary at this concluding stage of your manuscript. – **This has been removed**

Figures:

- major criticism of this paper is that there are too many stand alone figures and there is considerable redundancy between these. Please consider designing a figure that incorporates multiple lines (eg merge Figs 1 and 2). Also, your frequency tables for each of the interventions are redundant if you've already captured this in a previous figure. Strongly recommend rationalising your figures to just 3 or 4 in total, and use common axes to present the data more cleverly (eg you could plot mortality by week alongside some of the interventions). – **The figures have now been cut down to 4 in total**

Reviewer: 2

SERIOUS CONCERNS

There are different aims reported. In section abstract, the aim is “to collect physiotherapy interventions performed during the COVID-19 pandemic in a critical care setting to influence future practice and provide recommendations”, which are 3 aims: collecting PT data, influencing future, and providing recommendations, each requiring a different research design. In section introduction, you write “capture the Physiotherapy service delivered to our COVID-19 critical care cohort to determine incidence, frequency and timing of interventions”, and “This data may support future respiratory pandemic curricula, by identifying interventions which are likely to be …” and “this data adds to the global identification of Physiotherapy requirements”, and “may inform future practice recommendations.” These are a lot of different aims, incl. delivery and determination of PT, supporting curricula and inform practice recommendations. I strongly recommend to focus on reporting of “incidence and frequency of Physiotherapy interventions”, or else, and to report the same aim in sections abstracts and introduction. Please revise. – **Agreed the aim the abstract has now been changed.**

P4 L3: The part with the interventions is confusing and does not fit to the results. In section data collection, you write that interventions followed an A-E assessment (not defined, by the way) with focus on airway and breathing. Next is “assessment and limb care”, but for this project, it is interventions within 4 categories/domains. What is correct now? Please report your study in a stringent manner, and with appropriate methods. See also next point. – **Agreed A-E has now been defined and assessment and limb care has been added as an intervention.**

Interventions are reported in 4 domains (Secretion management, weaning, rescue therapy, functional rehab). If this is your main outcome, please report it in such a way. Please report these domains and the results in section abstract, and in section results, e.g. “of 2.248 interventions, #% (n=#) were domain a, #% (n=#) were domain b…” etc. **Agreed this has now been changed in the abstract and throughout the results section in the manuscript**

The report of “physical assessment and limb care” (p6, L33 pp) is confusing. You may report the most occurring intervention within each domain. The basic idea is: show the reader your design, describe the method so clearly that every reader can repeat the study, and report the main outcome. Report the outcome always in the same way, with identically domains and terms. Then it will be understandable. Please report in section results a) general data about the population, b) data of the 4 main domains, and c) other results of interest. Reduce the number of figures and tables.- **Agreed this has now been changed.**

Outcome “interventions per week” or “timing of interventions”: I do not understand this outcome. In section abstract/results you report “The weeks with the greatest number of interventions was week four with 373 interventions”. What does this mean? You started at a fixed date, April 1st, with an existing cohort of ICU patients, some were admitted days or weeks before the starting day, others at this day, and many more later. Hence, the number of interventions is not related to patients or the patients’ journey of their critical illness (what would have made sense: from day 1-7 after admission of a patient with Covid-infection, we made this and that, etc). The number of interventions was not related to the pandemic, because the pandemic has no fixed “starting point”; you just started during the main period of the pandemic, hence the timing of interventions was not even a chronologically answer to a crisis. So, what does the number of weeks and number of interventions mean? The number of interventions seems -from my view- to be influenced by cultural development and social adoption to an ongoing crisis, changing therapeutically concepts and other factors, but to see any relationship for this, you do not present relevant data. At all, I suggest to a) give good reasons why this measurement and outcome are relevant (maybe I misunderstand the approach), or b) delete this aspect from the whole manuscript incl. figures etc. **– Agreed this has now been reworded and the figure “interventions per week” has been taken out.**

Please add percentages to all numbers, where appropriate (#% (n=#)). – **This has now been changed**

You increased the staffing. Well done. In other countries, the PT to patient ratio is 1:9 or worse. How can you argue that this staffing ratio was necessary? How do you relate a staffing as recommended to a specific outcome? Contrary, you argued to need more staff, redeployed PTs and report a high mortality; a reader may conclude: “staffing with redeployed PTs will lead to increased mortality in ICU patients”. Why is this wrong? Can you make any conclusions about staffing and outcome? – **Thank you for this comment, we can appreciate that staffing may differ throughout different hospitals and different countries, this staffing ratio was a report of what staffing we were given during the pandemic not the staffing that was recommended. I can understand your point that the reader may come to conclusions however re-deployed staff had adequate training and we find the link between physiotherapy competency and mortality in ICU patients unfounded.**

MINOR CONCERNS

Page 2, Line 11: I am not a native speaker, but I guess that “COVID-19 outbreaks” are not able to speak, eat or see anything, even in English language. Please revise. – **This has now been changed**

P2 L13 please consider international readers; “NHS” is an abbreviation and should be spelled in full wording at first appearance. Same to CPAx, ACCU, etc etc etc, also in the main manuscript- **Agreed this has now been changed**

P2 L15 “multidisciplinary”: what do you mean? Physiotherapists belong to a profession, not a discipline. Dou you mean inter-professional, inter-disciplinary or both? Professions are defined as vocations requiring specialized knowledge and skills, e.g. physiotherapists, physician, nurses etc. Discipline is defined as academic branch of knowledge, e.g. cardiology, critical care, oncology… Please, review your manuscript for appropriate wording. – **This has now been changed**

P2 L28 you can suction a lot of things. You mean “endotracheal suctioning”?- **This has now been changed**

P2 L29: again, please consider international readers who may not know the CPAx. Please add a short information (such as “a scale measuring xy, from 0-50, with 50 indicating xy”) – **Agreed there is a full description of this in the methods section (page 4)**

P2 L45: I am sorry, but this project is not “unique”. Please delete. You did not report PT interventions “during the COVID 19 pandemic” but two months during the pandemic. Please revise. The data are not “vital”; data are data are data. – **This has now been changed.**

You do not report “frequency and timing” in the results, but just a sum; hence, you cannot draw any conclusion about frequency and timing; please a) add the data about frequency in section results, or b) replace it by sum of interventions. Please revise. – **The frequency has now been added to the results section.**

P2 L53: please schedule keywords in alphabetically order. – **This has now been changed**

P3 L 7 Well, maybe you prefer to introduce your study with a more general statement? **We are unsure how to address your concern and make a more general statement?**

P3, L2-17: please add some references to support your arguments/reports. **References are included within the introduction to support the statements**

P3 My apologies, but future readers and I do not know “our institution”. Please introduce the setting in short words- **This has now been changed.**

P4 L7 What do mean with “On 4th May 2020 a newly commissioned dedicated COVID-19 ACCU was opened within the hospital.” Was this ICU part of the research project or not? If it was part, how did it influence the workload and staffing? – **This has now been deleted.**

P4, L19 You mention “Forty-two Physiotherapists (Critical Care Physiotherapy specialists and redeployed staff)”, but a sentence later, “since less than 50% of the Physiotherapy team were critical care trained”. This is confusing. Hence, how many specialists and how many redeployed staff members were involved? – **This has now been changed.**

How many night calls were done? Did any PTs withdraw from work, due any reasons? I think these data are important for "future curricula" and else. – **This has now been added to the limitations.**

P4, L30 You mention PT's decision making and a "prioritization tool". The reviewer is (and future readers may be) from another country and is not familiar with ordering PT in UK. Who ordered physiotherapy? Did all patients receive an (automatic?) order, or does it depend on a personal judgement of PTs or physicians? Please describe in short words.- **Agreed this has now been explained.**

P5 L24 You mention statistics in a few words. In general, normal distributed data are reported as mean and standard deviation, and for metrical data tests such as t-tests etc can be used. Non-normal distributed data are reported as median and Interquartil Range (IQR), and other tests should be used for metrical data, such as Mann-Whitney U Test or other. Usually, data of length of stay etc. are not normal-distributed. I suppose the authors tested their data for distribution and used appropriate tests? Please add this information or revise the results.- **Thank you for your comment, this was an observational report with not statistics involved or statistical testing. It was purely a report of the numbers of interventions during the eight week time period**. **We have added the lack of statistic treatment of the data as a limitation.**

P5 L47 – P6 L16 Without any statistical tests, the comparison is … well, less informative and a "lower CPAx" can mean everything. I suggest to shorten the information. **Agreed this has now been deleted.**

P7 L31 usually, the first paragraph summarizes the design and the main results. It does not include any new information. – **This has now been deleted.**

P7, L43 – P11, L20 The discussion is much too length. Please reduce the text to: a) PT issues, b) the 4 domains, and continue with limits. – **This has now been changed.**

P11, L 20 there are much more limits, e.g. missing report of any illness severity and hence, no comparability to other populations, the likely recruitment bias of non-Covid patients, the missing statistics, missing data about the training of PTs etc. Please add more limits. – **This has been added.**

Please add a paragraph "conclusions". In case, the last paragraph P11 L45 is this, add the headline "conclusions" above. The last sentence "Although there was …" is a limit, not a conclusion, and should be placed there. – **This has been changed.**

Tables and figures: in general, tables and figures are self-explaining and include all information to understand and interpret them. Please add titles, foot notes incl. explanation of abbreviations, add descriptions of x/y axes, and avoid redundant information of figures vs manuscript of this information?- **This has now been changed.**
