# Supplementary material for: A single center observational study of the incidence, frequency and timing of critical care physiotherapy intervention during the COVID-19 pandemic
Source: J Intensive Care Soc. 2021 Feb 9;23(3):273–80. doi: 10.1177/1751143721991060 (PMC7873622; doi:10.1177/1751143721991060)
Supplement: Supplementary material [file sj-pdf-1-inc-10.1177_1751143721991060.pdf]

Dear Dr Corner,

Please convey to your reviewers our appreciation for the feedback received on the first submission of our manuscript. As you can imagine, completing this project and generating the energy required to produce a manuscript in these unprecedented times was challenging. The authorship team are all clinically facing with little allocated provision for quality activities such as these. Nevertheless, as a group we have learnt a significant amount from addressing the reviewers concerns and have revised the manuscript according to their guidance and recommendations. We have also taken this feedback into consideration for our consecutive data collection activities which will enhance our ability to report therapy intervention frequency in the future. We also acknowledge your suggestion to consider collaboration with other centres who may have similar therapy data and are making steps to join a collaborate Pan London group under your co-ordination. Our fledgling Therapy research interested group is encouraged by your support and enthusiastic about continuing to develop research active skills and writing for publication. In the meantime, we hope you will consider this revised manuscript for the Journal of Intensive Care. Changes in the manuscript are presented underlined for reader clarity. In addition, we have added comments to the reviewers notes in bold in the attached document.

Thank you,

Jessica Rich
